# Chloroplast genome draft assembly of *Falcataria moluccana* using hybrid sequencing technology

**DOI:** 10.1186/s13104-023-06290-6

**Published:** 2023-03-09

**Authors:** Vilda Puji Dini Anita, Deden Derajat Matra, Ulfah Juniarti Siregar

**Affiliations:** 1 Tropical Silviculture Program, Department of Silviculture, Faculty of Forestry and Environment, IPB University (Bogor Agricultural University), Bogor, Indonesia; 2 Department of Agronomy and Horticulture, Faculty of Agriculture, IPB University (Bogor Agricultural University), Bogor, Indonesia; 3 Department of Silviculture, Faculty of Forestry and Environment, IPB University (Bogor Agricultural University), Bogor, Indonesia

**Keywords:** Draft Chloroplast Genome, *Falcataria moluccana*, Long-reads, Short-reads

## Abstract

**Objectives:**

*Falcataria moluccana*, known locally as Sengon, is a fast-growing legume tree that is commonly planted in community forests of Java Island, Indonesia. However, the plantations face attacks of Boktor stem borer (*Xystrocera festiva*) and gall-rust disease (*Uromycladium falcatariae*) as major threats to its productivity. To control those pest and disease, it is necessary to grow resistant sengon clones, which are developed through tree improvement program, of which needs genetic and genomic information. This dataset was created to construct draft of sengon chloroplast genome and to study the evolution of sengon based on *matK* and *rbcL* barcode genes.

**Data description:**

Genomic DNA was extracted from leaf samples of one individual healthy tree in a private plantation. The DNA was sequenced using Illumina Novaseq 6000 (Novogen AIT, Singapore) for short-reads data, and MinION of Nanopore following manufacture’s protocols SQK-LSK110 for long-reads data. The 66,3 Gb short-reads and 12 Gb long-reads data were hybrid assembled and used to construct a 128.867 bp of *F*. *moluccana* chloroplast genome with a quadripartite structure, containing a pair of inverted repeats, a large single-copy and a small single-copy region. Phylogenetic tree constructed using *matK* and *rbcL* showed monophyletic origin of *F. moluccana* and other legume trees.

## Objective

*Falcataria moluccana*, locally known as Sengon, is main timber commodity in Indonesia, of which total production in 2019 reached 5.468.716,76 m^3^ [1], increased by 1.817.237,27 m^3^ from 2018 total production [2]. However, *F. moluccana* plantations have obstacles, especially from Boktor stem borer (*Xystrocera festiva)* and gall-rust (*Uromycladium falcatariae*) disease. These specific pest and disease also attack other tree species from Fabaceae family, such as from genus Acacia and Archidendron, with exception that in *F. moluccana* has caused more severe losses [3]. Since effective control methods are not available, it is necessary to develop resistant *F. moluccana* from these pest and disease.

*F. moluccana* improvement program has been conducted; however, progress is slow considering the complexity of the resistant traits. In such case genomic approach could assist the selection program by providing information on important genes related to resistance to pests and diseases. Some genes related to resistance to biotic and abiotic stress, as well as adaptation could be located in the cytoplasm, such as in the chloroplast genome [4]. The host range of Boktor stem borer pest and gall-rust disease among trees from Fabaceae family posed an interesting evolutionary relationship among those tree species in the Fabaceae family. Chloroplast genome is relatively small in size and very conservative that it becomes popular subject for studying genetic and evolutionary relationship among plant species [5]. This study aimed at constructing a complete and high quality of *F. moluccana* chloroplast draft genome utilizing the advance of sequencing technology such as Next-generation Sequencing (e.g. Illumina) and Third-generation Sequencing (e.g. Oxford Nanopore) with bioinformatics approach [6], also to find out the evolutionary relationship of *F. moluccana* with several other tree species from Fabaceae family using *matK* and *rbcL* genes, which are commonly used in DNA barcoding.

**Table 1:** Overview of data files/data sets

Label	Name of data file/data set	File types (file extension)	Data repository and identifier (DOI or accession number)
*Data file 1*	*Statistic of Short-read and Long-read Data of Sengon (Falcataria moluccana)*	*Document file (.docx)*	*Figshare *10.6084/m9.figshare.21626951.v1 [21]
*Data file 2*	*Circular map of F. moluccana chloroplast genome*	*Picture file (.PNG)*	*Figshare *10.6084/m9.figshare.21627005.v1 [22]
*Data file 3*	*List gene on sengon chloroplast genome*	*Document file (.docx)*	*Figshare *10.6084/m9.figshare.21626993.v1 [23]
*Data file 4*	*Phylogenetic tree of matK and rbcL*	*PNG file in compressed file (.rar)*	*Figshare *10.6084/m9.figshare.21627014.v1 [24]
*Data set 1*	*Raw Short-reads data*	*Fastq files (.fastq)*	DNA Data Bank of Japan (DDBJ) accession number DRA012508 (https://trace.ddbj.nig.ac.jp/DRASearch/submission?acc=DRA012508) [25]
*Data set 2*	*Raw Long-reads data*	*Fastq files (.fastq)*	DNA Data Bank of Japan (DDBJ) accession number DRA015209 (https://trace.ddbj.nig.ac.jp/DRASearch/submission?acc=DRA015209) [26]

## Data description

Genomic DNA was extracted from 400 mg fresh leaf samples using CTAB method from [7] with modifications. The leaves were collected from one 7 years-old individual healthy tree, grown at a private plantation in Cikarawang Village, Bogor, West Java. The quality of extracted genomic DNA was evaluated using agarose gel electrophoresis. The purity of the genomic DNA was assessed using NanoPhotometer NP80 Implen and the quantity was measured using Qubit 1.0 Fluorometer with Qubit dsDNA BR (Broad-Range) Assay Kit. Short-reads sequencing was done using Illumina Novaseq 6000 (Novogen AIT, Singapore), while long-reads sequences were obtained using MinION from Nanopore, following manufacture’s protocols SQK-LSK110. Data can be accessed from DNA Data Bank of Japan (DDBJ) with accession number DRA012508 for short-reads data (Dataset 1) [25] and DRA015209 for long-reads data (Dataset 2) [26].

Hybrid chloroplast genome assembly was performed using the pipeline from http://github.com/asdcid/Chloroplast-genome-assembly [8]. The pre-assembly was performed by quality check, following the script from http://github.com/asdcid/Chloroplast-genome-assembly/tree/master/1_pre_assembly. Short-reads data was quality checked using FASTQC [9] and trimmed using BBDukv37.31 [10]. Quality check for long-reads data was also done using FASTQC program. Adapter trimming was performed using Porechop v0.2.1 [11] while quality trimming was done using NanoFilt v1.2.0 [12]. The trimming result were double checked using FASTQC. From this pre-assembly step, the total bases of long-reads data were reduced from 12Gb to 11Gb, while for short-reads data was reduced from 66,3 Gb to 63,4 Gb (Data file 1). These clean-reads were aligned to the reference NC_047364.1 (*F. moluccana*) using Bowtie v2.2.6 [13] for short-reads and Blasrv5.1 for long-reads [14].

Chloroplast-mapped reads were assembled using Unicycler v0.3.1 [15] and corrected using SPAdes in Unicycler with default settings from http://github.com/asdcid/Chloroplast-genome-assembly/tree/master/2_assembly. Afterwards, script from http://github.com/asdcid/Chloroplast-genomeassembly/tree/master/3_post_assembly was performed for post-assembly step. All contigs are combined into a single contigs with the same structure against used reference using Mummer v2.23 [16] and Pilon v1.20.1 to polish the data [17]. Draft of chloroplast contig were annotated using GeSeq [18] towards all Fabaceae reference in NCBI RefSeq and visualized using OGDRaw in MPI-MP Chlorobox [19] (Data file 2). The chloroplast genome encoded 95 genes, composed of 27 tRNA genes, 1 rRNA gene, and 67 protein coding genes (Data file 3). Phylogenetic analysis reconstruction was performed using MEGAX (Molecular Evolutionary Genetic Analysis) v10.2.2 [20] with Maximum Likelihood method, Tamura-3 model and bootstrap value of 10.000 replication. For the phylogenetic analysis *Intsia bijuga* (NC_047336.1) was used as an outgroup. Based on phylogenetic analysis using *matK* and *rbcL* gene markers, the constructed phylogenetics trees indicated a monophyletic topology. The phylogenetic tree using *matK* showed 3 groups (Data files 4, Fig. 2A), of which *F. moluccana* in this study are in the same clade with *Archidendropsis granulosa* in the second group and separated from other *F. moluccana* accessions. In the case of *rbcL* marker, the phylogenetic tree formed 9 groups (Data files 4, Fig. 2B), of which the *F. moluccana* studied are placed in the same group no. 9 with other *F. moluccana* accessions.

## Limitations

This study used leaves samples from one individual tree accession in a private plantation, with unknown origin. The tree selected shows resistance to pest and disease attacks.

## Data Availability

The data described in this Data note can be freely and openly accessed on DNA Data Bank of Japan (DDBJ) with accession number DRA012508, DRA015209, and figshare. Please see Table 1 and references list [21–26] for details and links to the data.
